# Patient‐Derived iPSC‐Cardiomyocytes Reveal Subclinical Cardiomyocyte Dysfunction Associated With the *CAV3* N‐Terminal Variant p.Ala46Thr

**DOI:** 10.1155/humu/4061668

**Published:** 2026-09-22

**Authors:** Junyi Wang, Xintong Zhu, Yaqiong Li, Limeng Dai, Xiaoyin Tan, Jia Li, Hong Guo

**Affiliations:** ^1^ Department of Medical Genetics, College of Basic Medical Science, Army Medical University (Third Military Medical University), Chongqing, China, tmmu.edu.cn; ^2^ Department of Gynecology and Obstetrics, Southwest Hospital, Army Medical University (Third Military Medical University), Chongqing, China, tmmu.edu.cn; ^3^ NHC Key Laboratory of Birth Defects and Reproductive Health (Chongqing Key Laboratory of Birth Defects and Reproductive Health), Chongqing Population and Family Planning Science and Technology Research Institute, Chongqing, China

**Keywords:** calcium imaging, *CAV3* gene, iPSC-CMs, p.Ala46Thr, subclinical cardiomyocyte dysfunction

## Abstract

The *CAV3* p.Ala46Thr mutation is previously associated with skeletal muscle damage only, with its myocardial impact and long‐term cardiac risks unclear. This study is aimed at investigating whether it induces subclinical myocardial damage at the cellular level and explore its mechanism using a patient‐specific iPSC‐CMs model. To achieve this, peripheral blood from a proband carrying the *CAV3* c.136G>A (p.Ala46Thr) mutation but only clinical manifestations of skeletal muscle damage was collected. Subsequently, a patient‐derived iPSC line was established and directionally differentiated into iPSC‐CMs. IF and TEM were then conducted to observe the structure of cardiomyocytes, the arrangement of sarcomeres, and the subcellular localization of CAV3 protein. Additionally, laser confocal calcium imaging technology was used to analyze the basic electrophysiological rhythm of cardiomyocytes and their response to fenoterol. As a result, the mutant iPSC‐CMs model was successfully constructed. Compared with controls, mutant iPSC‐CMs exhibited a normal cellular structure, but notably, the CAV3 protein exhibited abnormal aggregation around and inside the nucleus, resulting in a loss of membrane localization. Furthermore, functional assays showed that the mutant iPSC‐CMs had a significant decrease in basic beating frequency and an irregular rhythm. After stimulation with fenoterol, the increases in beating frequency and rhythm regularity were significantly less pronounced than those in controls. In conclusion, this study is the first to demonstrate that human cardiomyocytes from carriers of the *CAV3* p.Ala46Thr mutation without any cardiac symptoms clinically exhibit an abnormal localization of CAV3 protein and definite altered spontaneous activity and calcium‐handling behavior. This suggests that the mutation can induce subclinical myocardial damage, and the carriers should be considered as a group with potential risk of heart disease. Overall, this study provides a novel experimental basis for the early warning and accurate treatment of *CAV3*‐related diseases on the site of clinics.

## 1. Introduction

Caveolin‐3 is encoded by the *CAV3* gene (OMIM 601253) and serves as a scaffold protein for caveolar structures in the cell membrane of skeletal and cardiac muscle [1]. In addition to participating in membrane budding and caveolar endocytosis, it is also involved in signal transduction as a key component regulating calcium homeostasis and adrenergic receptor responses [2]. Mutations in *CAV3* are associated with a group of hereditary disorders affecting both skeletal and cardiac muscle, collectively termed caveolinopathy [3]. These include limb‐girdle muscular dystrophy Type 1C, distal myopathy, and episodes of rhabdomyolysis, often accompanied by elevated serum creatine kinase (CK) levels.

Depending on the location of mutations within the protein domains, the clinical phenotypes of *CAV3* mutations show a distinct pattern. Mutations in the transmembrane domain (e.g., p.T78M and p.A85T) typically present with significant cardiac manifestations such as dilated cardiomyopathy, hypertrophic cardiomyopathy, or long QT syndrome, resulting from direct impairment of caveolar membrane integration function [4]. In contrast, some mutations in the N‐terminal scaffolding domain (e.g., p.R26Q) are mostly reported to cause only mild skeletal muscle pathology without definite cardiac involvement [5]. This observation formed the previous view that mutations in the N‐terminal scaffolding domain predominantly induce skeletal muscle damage with minimal cardiac risk.

However, this view is challenged by mutations within the highly conserved FEDVIAEP sequence (Amino Acids 41–48) in the N‐terminus [6]. This sequence is a critical site where Caveolin‐3 binds to the cardiac sodium channel (Nav1.5) and regulates its function [7]. Specifically, the p.Ala46Val mutation within this sequence has been clearly identified as a cause of severe cardiac phenotypes, including cardiac conduction block and dilated cardiomyopathy [8]. The p.Ala46Thr (c.136G>A) mutation, which differs from p.Ala46Val by one amino acid, also localizes to this core functional domain and induces a similarly nonconservative amino acid change [9]. This mutation has been first reported in patients with skeletal muscle diseases, which also showed that it prevents the localization of Caveolin‐3 to the plasma membrane in a dominant negative fashion [10]. Moreover, p.Ala46Thr has been identified in several independent pedigrees and is associated with distal myopathy, characterized by hand muscle atrophy, muscle weakness, and markedly elevated serum CK levels [11]. However, systematic studies on whether A46T also confers cardiac risk, especially comparative functional studies with p.Ala46Val, remain lacking to date.

This comparison raises a critical scientific question. Whether p.Ala46Thr exerts similar but potentially more subtle or late‐onset deleterious effects on cardiomyocyte (CM) function and whether the absence of cardiac manifestations in carriers is due to subclinical disease or compensatory mechanisms, remains to be determined.

Induced pluripotent stem cell‐derived cardiomyocytes (iPSC‐CMs) have emerged as an important platform for addressing this question. This model retains the complete genetic background of patients and generates disease‐relevant CMs in vitro, making it particularly suitable for recapitulating early electrophysiological and contractile dysfunction [12]. It has demonstrated unique advantages in mechanistic studies of various hereditary cardiomyopathies.

Therefore, this study identified a proband harboring the *CAV3* p.Ala46Thr mutation with no overt cardiac manifestations during long‐term clinical follow‐up. By generating a patient‐specific iPSC‐CM model, this study is aimed at directly addressing the following questions: whether the mutant Caveolin‐3 protein exhibits aberrant subcellular localization; whether p.Ala46Thr elicits alterations in electrophysiological properties; and whether it impairs CM responsiveness to *β*‐adrenergic receptor stimulation. Based on the known mechanisms of p.Ala46Val, this study intends to systematically assess the potential myocardial injury risk of p.Ala46Thr at the cellular functional level, even in the absence of clinical cardiac symptoms and thereby provide experimental evidence for the long‐term clinical management of carriers.

## 2. Methods

### 2.1. Clinical Samples and Detection Methods

#### 2.1.1. Clinical Sample Collection

This study was performed with the approval of the Ethics Committee of Army Medical University, Chongqing, China, Ethics Approval Number 2022‐10‐01. This study included a family with inherited cardiomyopathy carrying the *CAV3* p.Ala46Thr mutation (38 family members: eight affected patients and 30 healthy controls). All family members signed the informed consent form. Clinical data, including medical history, physical examination, and ECG, were collected from the neurology department of the Second Affiliated Hospital of Army Medical University. Long‐term follow‐up was also performed. Peripheral venous blood (2 mL) was collected from all family members using EDTA as an anticoagulant.

#### 2.1.2. Sanger Sequencing

Genomic DNA was extracted from 200 *μ*L peripheral blood using the TIANamp Genomic DNA kits (Tiangen, China) and then stored at −20°C. The concentration and purity of genomic DNA were assessed using a NanoDrop 1000 Ultra‐Micro Spectrophotometer (Thermo Fisher Scientific). Sequential genetic testing was performed, including variant screening from whole‐exome sequencing data, comparison with polymorphism databases, Sanger sequencing validation, cosegregation analysis, and prediction of mutation deleteriousness, to identify the candidate pathogenic variant in this family. The primers for amplifying the *CAV3* p.Ala46Thr region were 5 ^′^‐CACACACCCAAAAGCTTGAGA‐3 ^′^ (forward) and 5 ^′^‐GCTCTTAATGCATGGCACCA‐3 ^′^ (reverse).

PCR amplification was performed in a total volume of 50 *μ*L containing 25 *μ*L of 2×T5 Super PCR Mix, 18 *μ*L of ultrapure water, 2 *μ*L each of forward and reverse primers, and 3 *μ*L of DNA (Tsingke Biotechnology, Beijing). The PCR program included predenaturation at 98°C for 3 min, 35 cycles (denaturation at 98°C for 10 s, annealing at 60°C for 10 s, and extension at 72°C for 17 s, and the extension time was calculated based on the length of the product of 281 bp at a conventional rate of 1 kb/min) and a final extension at 72°C for 5 min. The PCR products were then stored at 4°C for later use.

The PCR products were sequenced using a mix of normal and modified nucleotides (dNTPs and ddNTPs). The ddNTPs terminated the DNA strand synthesis at random points. The resulting fragments were separated by size using capillary electrophoresis, with each fragment labeled with a different fluorescent dye. Sequence data were analyzed with SnapGene software (GSL Biotech LLC).

Cosegregation of the variant with the family phenotype was analyzed, and pathogenicity was evaluated in accordance with the American College of Medical Genetics and Genomics (ACMG) guidelines [13].

### 2.2. Establishment of Patient‐Derived iPSC Model

#### 2.2.1. Reprogramming of Erythroid Progenitors Into iPSCs

Peripheral blood was collected from the proband and healthy control (an unaffected sister III‐5 who tested negative for the *CAV3* mutation). Mononuclear cells were isolated and induced to differentiate into erythroid progenitor cells (EPCs) using EPC‐specific induction medium. EPCs were introduced with reprogramming factors by electroporation using the Epi5 Episomal iPSC Reprogramming Kit. Transfected cells were plated onto Matrigel plates, grown in EPC medium for 3 days, and then the media was changed to ReproTeSR Reprogramming Medium for maintenance culture. Around Days 10–25, the iPSC colonies with typical iPSC morphology were selected and then subcultured and expanded for use in further studies.

#### 2.2.2. Karyotype Analysis

At 50%–60% confluency in six‐well plates, cells were treated with 200 ng/mL colcemid (2 h), washed, dissociated, and centrifuged (1000 g, 7 min). The pellet was resuspended in 8 mL prewarmed (37°C) 0.075 M KCl (25 min, 37°C water bath). After adding 2 mL fixative (methanol : acetic acid = 3 : 1, fresh), cells were prefixed (15 min, RT), centrifuged (1000 g, 7 min), and resuspended in 8 mL fixative (15 min, RT), and then centrifuged again. A second fixation with 8 mL fixative was performed overnight at RT. After centrifugation, a small volume was retained, mixed, dropped onto prechilled tilted slides, and flame‐dried. Slides were stained with Giemsa (1:10 in D‐PBS, 10 min), rinsed, and examined.

#### 2.2.3. Pluripotency Assessment by Immunofluorescence (IF)

Cells grown on Matrigel‐coated coverslips in 12‐well plates were fixed with 4% paraformaldehyde (15 min, RT), washed (D‐PBS, 15 min), permeabilized with 0.1% Triton X‐100 (5 min), and blocked (1 h, RT). Primary antibodies (OCT4, SOX2 1:500; Nanog 1:250) were applied overnight at 4°C. After washing, secondary antibodies (Alexa Fluor 488, 647, Cy3, all 1:500) were added (2 h, 37°C, dark). Nuclei were stained with DAPI (1:5, 30 min, RT, dark). Coverslips were mounted and imaged using a confocal microscope. For each group of staining, at least three slides were selected. For each slide, five fields of view were randomly chosen, and at least 50 cells of each marker were counted in each field of view by using ImageJ.

#### 2.2.4. Microbiological Testing

One milliliter of iPSC supernatant was evenly spread onto Sabouraud agar plates and sheep blood agar plates. Fungal cultures were incubated at 25°C and bacterial cultures at 37°C. The plates were observed daily for 5 days, and the results were recorded. The mycoplasma PCR test kit (Cellapy Biotechnology, CA6101024) was used to check for any mycoplasma contamination by following the manufacturer′s instructions.

#### 2.2.5. Mutation Gene Testing (Sanger Sequencing)

Approximately 5 × 10^6^ cells (patient and control iPSCs) were lysed with 200 *μ*L buffer GA and 20 *μ*L proteinase K. Genomic DNA was extracted using a kit (Tiangen, Beijing) following the manufacturer′s protocol. PCR amplification and sequencing were performed as described in Section 2.1.

### 2.3. Establishment of Patient‐Derived iPSC‐CMs Model

#### 2.3.1. Differentiation of iPSCs Into iPSC‐CMs

iPSCs were differentiated into iPSC‐CMs via a small‐molecule temporal modulation approach. CHIR 99021 (4 *μ*M) was added to RPMI 1640/B‐27 medium (without insulin) to activate the Wnt signaling pathway and then incubated for 48 h. Replaced with fresh media after 24 h, IWR‐1 (5 *μ*M) was added to inhibit the Wnt pathway for > 48 h. Then, the medium was changed to complete B‐27 medium containing insulin for maintenance culture. Spontaneous beating cell clusters could be seen from Day 9 of differentiation. Metabolic purification was performed using glucose‐free medium supplemented with sodium lactate from Days 13–16.

#### 2.3.2. Measurement of iPSC‐CMs Maturity by IF

Cells grown on Matrigel‐coated coverslips in 12‐well plates were fixed with 4% paraformaldehyde (15 min, RT), washed (D‐PBS, 15 min), permeabilized with 0.1% Triton X‐100 (5 min), and blocked (1 h, RT). Primary antibodies (cTnT 1:500; *α*‐actin 1:200) were applied overnight at 4°C. After washing, secondary antibodies (647, Cy3, all 1:500) were added (2 h, 37°C, dark). Nuclei were stained with DAPI (1:5, 30 min, RT, dark). Coverslips were mounted and imaged using a confocal microscope.

ImageJ software was used to analyze morphological differences in IF images of differentiated CMs from patients and control groups. For each group, 10 independent fields were randomly selected; one to two cells were counted in each field. The total number of cells analyzed in each group was no less than 10. The differences in sarcomere length of differentiated CMs were measured by *α*‐actin IF staining and ImageJ software. The expression of the *TNNT2*‐encoded protein (cTnT) in iPSC‐CMs was observed by IF.

### 2.4. Detection of Molecular Mechanisms

#### 2.4.1. The Expression of CAV3 Protein in iPSC‐CMs by IF and Western Blot (WB)

Cells grown on Matrigel‐coated coverslips in 12‐well plates were fixed with 4% paraformaldehyde (15 min, RT), washed (D‐PBS, 15 min), permeabilized with 0.1% Triton X‐100 (5 min), and blocked (1 h, RT). Primary antibodies (CAV3 1:100, abcam, ab289544) were applied overnight at 4°C. After washing, secondary antibodies (488 1:500) were added (2 h, 37°C, dark). Nuclei were stained with DAPI (1:5, 30 min, RT, dark). Coverslips were mounted and imaged using a confocal microscope.

Cells were lysed in RIPA buffer with protease/phosphatase inhibitors, sonicated, and centrifuged (7500 g, 5 min, 4°C). Protein concentration was determined by BCA assay, adjusted to 1 *μ*g/*μ*L, mixed with 5× loading buffer, and boiled at 100°C for 5 min. Protein samples (10 *μ*g/lane) were resolved by 10% SDS‐PAGE (60 V for 30 min, and then 120 V for 60 min), transferred onto a methanol‐activated PVDF membrane (25 V, 2,500 mA, 7 min), and blocked with 5% nonfat milk for 1 h. The membrane was incubated overnight at 4°C with rabbit anti‐CAV3 (1:1,000, abcam, ab289544) and mouse anti‐GAPDH (1:10,000), followed by HRP‐conjugated goat antirabbit secondary antibody (1:10,000) for 2 h at room temperature. After TBST washes, signals were detected by ECL and normalized to GAPDH for statistical analysis. For IF analysis, 11 randomly selected fields were examined per group, each containing one to three cells.

#### 2.4.2. Transmission Electron Microscopy (TEM) Detection

The medium was removed, cells were washed twice with D‐PBS, and then dissociated with Accutase. The digested cells were collected into a 1.5 mL tube, centrifuged at 1000 g for 5 min, after which the supernatant was discarded. One milliliter of cold fixative (glutaraldehyde) was added along the inner wall of the tube and fixed it at 4°C overnight. The central laboratory of the Army Medical University completed the subsequent processing and observation.

#### 2.4.3. Detection of CM Rhythm

The matrix gel was laid out in the special cell culture dish for confocal microscopy. The cells were digested into single cells using Accutase and then inoculated into the gel. The cells were cultured for 24 h to allow them to adhere to the surface. The adherent cells were washed twice with D‐PBS. The calcium ion staining agent Fluo‐4 was diluted in cell culture medium (without serum) to a final concentration of 4 *μ*M. The prepared solution was then added to the cell culture dish, and the cells were placed in a 37°C incubator for 30 min. The culture medium containing the dye was removed, the cells were washed twice with D‐PBS, and 1 mL of culture medium without dye was added. This can be observed and recorded under a confocal microscope at room temperature (21°C). Dynamic fluorescence recordings were conducted using the time series mode of the confocal microscope. The frame interval was set at approximately 1.27 s (with a sampling frequency of approximately 0.79 Hz), and the total recording duration for a single field of view was 15 min. Once the rhythm of the cell fluorescence intensity changes stabilizes, 1 *μ*L of fenoterol (final concentration 10 *μ*M) was carefully added to the dish, and recording of the changes in fluorescence intensity was continued for the remaining duration. Data analysis was conducted after the shooting was completed. The average fluorescence intensity of the untreated cells during the resting period was taken as F_0_, and the absolute fluorescence intensity at each time point was F. The relative fluorescence change value *Δ*F/F_0_ = (F − F_0_)/F_0_ was calculated. A total of 13 CMs were randomly selected from each group from at least three independent experiments for calcium imaging recording and analysis.

### 2.5. Data Analysis Methods

Statistical analyses and data processing were performed using SPSS 26.0 software, statistical graphs were generated using GraphPad Prism 9.0 software, and IF images were processed with ImageJ software. Continuous data were presented as mean ± standard deviation (mean ± SD), with at least three independent replicates per group. An unpaired *t*‐test was used to compare data between the mutant and control groups. The chi‐square test was used for categorical data. The original data of CM rhythm indicators were obtained from Zen software, and the difference between groups was analyzed after calculating *Δ*F/F_0_; *p* < 0.05 was considered to have reached statistical significance marked as  ^∗^ *p* < 0.05,  ^∗∗^ *p* < 0.01,  ^∗∗∗^ *p* < 0.001.

## 3. Results

### 3.1. Clinical Characteristics and Genetic Diagnosis of the Proband

**Figure 1:**
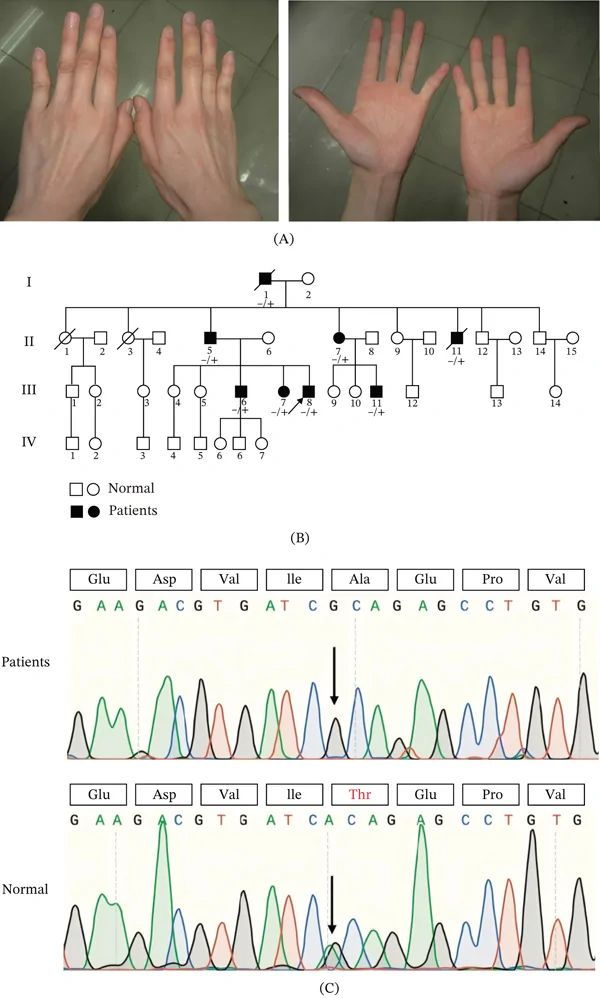
Pedigree and sequencing results of the *CAV3* gene mutation. (A) The clinical manifestations of the proband. (B) The pedigree of the patient; black indicates clinical manifestation, diagonal line indicates death, and the arrow indicates the proband. (C) The results of Sanger sequencing, with normal family members as the normal control.

A male patient with skeletal myopathy who carries the *CAV3* mutation and his family members were recruited into this study, and the core clinical data, examination data, and follow‐up data of the patient are shown in Figure 1.

The patient was an 18‐year‐old male who presented with a complaint related to hand muscle atrophy with weakness >3 years. Physical examination: active tendon reflexes in both upper limbs, negative Hoffmann sign, muscle atrophy of both hands (including bilateral interosseous muscles, thenar and hypothenar muscles, and bilateral lumbrical muscles), no obvious sensory impairment, plump limb muscles, and normal muscle strength. Serum CK level detection results of three consecutive tests were 3197 IU/L, 1659 IU/L, 3651 IU/L, all above normal reference value 26–174 IU/L, indicating that there was definite skeletal muscle involvement, and electromyography also showed myogenic damage. Electrocardiogram (ECG) showed sinus rhythm, normal cardiac function, and no organic cardiac changes, indicating no current myocardial ischemia and no electrical activity abnormalities.

Genetic and other examination results were definite: Proved by Sanger sequencing, the patient had heterozygous *CAV3* c.136G>A (p.Ala46Thr) mutation, which was recorded in the ClinVar database (Variation ID: 8281, Accession: VCV000008281.52), and met the ACMG guideline criteria (PS1 + PM1 + PM2 + PP1 + PP5) for pathogenicity rating as “Pathogenic (P)” and cosegregated with the skeletal muscle phenotype in the family. Cervical spine plain MRI showed a straight physiological curvature of the cervical spine, with herniation of the C3/4 and C4/5 intervertebral discs, but no spinal cord or nerve root compression, thus ruling out hand weakness due to cervical spine lesion and further supporting that the skeletal muscle lesion was due to the *CAV3* mutation.

An 11‐year follow‐up study was conducted. During the follow‐up, the proband was followed up by phone. They gave a subjective report that they only had hand weakness without any aggravation, no daily activity limitations, and no cardiac discomfort like chest tightness, shortness of breath and palpitations. The result showed that the patients′ skeletal muscle symptoms did not improve, the hypothenar muscle atrophy of both sides showed no obvious regression, and the CK level remained between 1500–3800 IU/L. The other seven family members carry the same mutation, among which five are alive, one died from an unknown cause (I‐1), and one died from suspected heart disease (II‐11, based on family statement, without confirmatory evidence).

### 3.2. Successful Establishment of the Induced Pluripotent Stem Cell Model From Patients With the *CAV3* p.Ala46Thr Mutation

**Figure 2:**
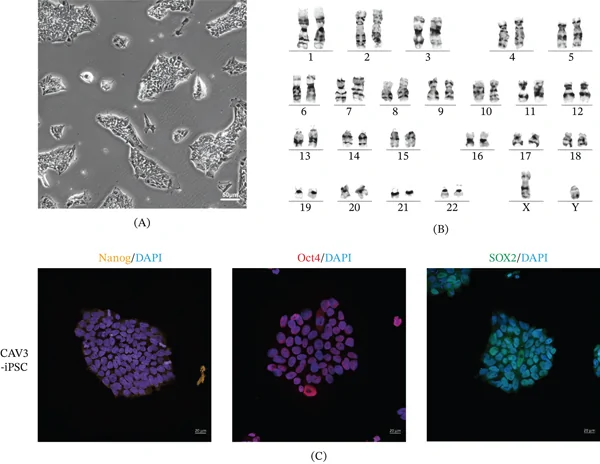
Identification of the *CAV3*‐iPSC cell line. (A) Morphology of iPSCs observed under microscope at 200x magnification. (B) Karyotype distribution of cells. (C) Expression of Nanog, OCT4, and SOX2 (400x).

EPCs were isolated from the patient′s peripheral blood and reprogrammed to generate a patient‐derived *CAV3*‐iPSC cell line. Under bright‐field microscopy, the cells grew as compact, tightly packed aggregates with visible nucleoli. Furthermore, the colony edges were smooth, and no differentiated cells were observed (Figure 2A). Karyotype analysis of metaphase spreads revealed intact chromosomes with no numerical or structural abnormalities (Figure 2B). IF staining, examined under a laser confocal microscope, confirmed that the established iPSCs expressed the pluripotency markers OCT4, SOX2, and Nanog (Figure 2C). Microbial testing of the culture supernatant at 120 h showed no bacterial or fungal growth. Sanger sequencing showed that the *CAV3*‐iPSC cell line had the pathogenic heterozygous mutation *CAV3* c.136G>A (p.Ala46Thr), which was consistent with the previous results of whole‐exome sequencing and Sanger sequencing.

### 3.3. Successful Establishment of the Patient‐Derived iPSC‐CMs Model

**Figure 3:**
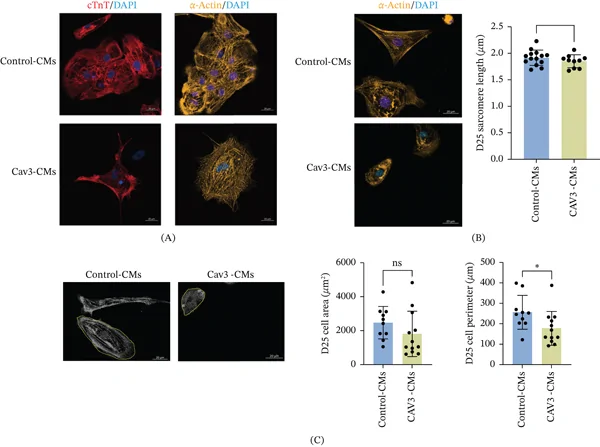
Detection of CAV3‐CMs structure. (A) Immunofluorescence staining of cTnT and *α*‐actin, red for cTnT, yellow for *α*‐actin, blue for cell nucleus. (B) ImageJ software was used to measure sarcomere length in micrographs of differentiated cardiomyocytes. The yellow straight lines indicate the regions subjected to measurement. The bar graphs represent the statistical analysis of the sarcomere length measurements. (C) ImageJ software was employed to measure cell perimeter and cell area in micrographs of differentiated cardiomyocytes. The yellow lines trace the cell perimeter, and the areas enclosed by the yellow lines denote the cell areas. The bar graphs depict the statistical results of the sarcomere length measurements.

*CAV3*‐iPSCs were induced to differentiate into CMs, and detection was performed on the 25th day after screening. IF staining for cardiac muscle proteins cTnT and *α*‐actin showed no significant differences in protein distribution and cell structure between *CAV3*‐CMs and normal controls (Figure 3A). The morphological quantitative analysis of IF images was conducted using ImageJ software. The analysis revealed that the sarcomere length was 1.92 ± 0.14 * μ*m in the control‐CMs group and 1.85 ± 0.12 * μ*m in the CAV3‐CMs group, with no statistically significant difference between the two groups (*p* ≥ 0.05) (Figure 3B). The cell area was 2465.16 ± 962.68 * μ*m^2^ in the control‐CMs group versus 1804.98 ± 1335.16 * μ*m^2^ in the CAV3‐CMs group, showing no statistically significant difference (*p* ≥ 0.05) (Figure 3C). The cell perimeter was 255.78 ± 83.20 * μ*m for the control‐CMs group and 177.52 ± 82.72 * μ*m for the CAV3‐CMs group; the difference between the two groups was statistically significant ( ^∗^ *p* < 0.05) (Figure 3C).

### 3.4. Functional Verification of iPSC‐CMs

#### 3.4.1. TEM Detection Showed That the CAV3 p.Ala46Thr Mutation Has No Significant Effect on CM Structure

**Figure 4:**
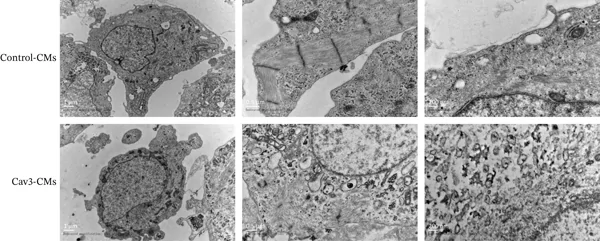
Representative TEM images of control and *CAV3* p.Ala46Thr mutant iPSC‐CMs. Representative TEM micrographs illustrating the subcellular morphology in both groups, with a focus on caveolar structures. The overall ultrastructural features were comparable between groups. Scale bars: 1 *μ*m, 0.5 *μ*m, and 200 nm as indicated.

*CAV3*‐iPSCs were induced to differentiate into CMs, and detection was performed on the 25th day after screening. TEM analysis showed that iPSC‐CMs from both the mutant and control groups displayed canonical ultrastructural characteristics of CMs. The myofibrils of the two groups of cells were arranged in a regular pattern, clear Z‐lines, and normal‐shaped mitochondria with complete cristae structure. No obvious breaks or vacuolization changes were observed in the cell membranes and other organelles (Figure 4). Both groups of cells showed the typical caveolae structure, which was characterized by an *Ω*‐shaped or flask‐shaped indentation. By comparison, from those of the control group, the morphology, size, and distribution density along the plasma membrane of the mutant group were not significantly different. This result showed that the *CAV3* p.Ala46Thr mutation did not significantly affect the conventional ultrastructure of iPSC‐CMs and the caveolae‐associated vesicular structures under basal culture conditions.

#### 3.4.2. The CAV3 Protein Was Expressed Aberrantly in the CAV3 p.Ala46Thr Mutation Group

**Figure 5:**
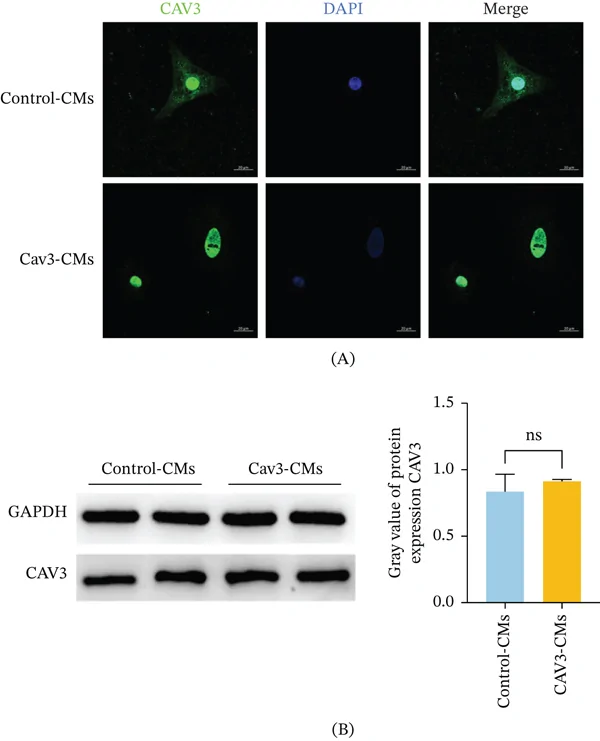
Detection of *CAV3*‐CMs structure and localization and quantification of CAV3 protein. (A) Localization detection of CAV3 protein by immunofluorescence staining, green for CAV3 protein, blue for cell nucleus. (B) Detection of CAV3 protein levels in *CAV3*‐CMs and normal cardiomyocyte controls by WB, GAPDH as the internal reference; the bar chart is the statistical graph of data analysis.

*CAV3*‐iPSCs were induced to differentiate into CMs, and detection was performed on the 25th day after screening. The control group was distributed in the cytoplasm and cell membrane of CAV3 protein, and there was no distribution in the cell membrane in the case group, only CAV3 aggregate in and around the nucleus (Figure 5A). Total proteins were obtained from the *CAV3*‐CMs and normal control group, and the expression of the CAV3 protein was detected. No obvious difference in CAV3 protein level was observed (Figure 5B). A total of 23 cells were observed in the mutant group, among which 20 cells showed abnormal distribution of the CAV3 protein (86.96%). In the control group, 22 cells were observed, and the abnormal distribution of CAV3 protein was not found in any of them.

#### 3.4.3. The CAV3 p.Ala46Thr Mutation Impairs CM Rhythm and Responsiveness to Fenoterol

**Figure 6:**
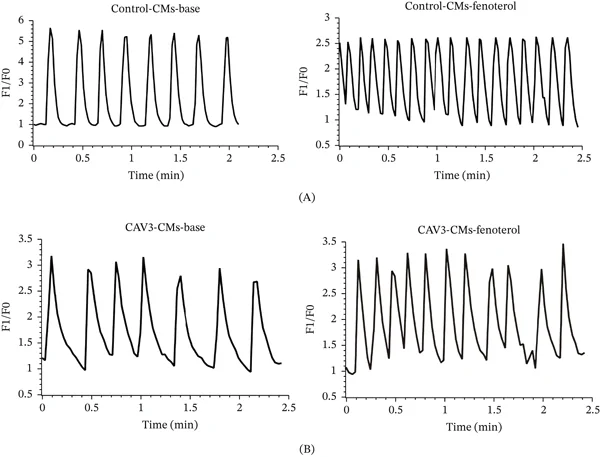
Rhythm detection of *CAV3*‐CMs. (A) Resting rhythm of cardiomyocytes of the normal control group and the rhythm changes after 1 *μ*L fenoterol was added to the medium (*n* = 13). (B) Resting rhythm of cardiomyocytes of the *CAV3*‐CMs group, and the rhythm changes after 1 *μ*L fenoterol was added to the medium (*n* = 13).

*CAV3*‐iPSCs were induced to differentiate into CMs, and detection was performed on Day 30 after myocardial maturation screening. Calcium imaging detection of CMs was performed using laser confocal microscope, with the intracellular calcium ions labeled with calcium sensitive fluorescent probe Fluo‐4. The intensity of green fluorescence is proportional to Ca^2+^ concentration. The results showed that in the control‐CMs group without fenoterol, four rhythmic beating events were detected within 60 s (BPM: 4.0). CV was 1.87%, indicating that the beating rhythm was highly regular. After using the *β*‐adrenergic receptor agonist fenoterol, the pulse frequency in the control group increased to seven beats within 60 s (BPM: 7.0), and the CV further decreased to 0.97%, indicating that adrenergic stimulation can increase the beating rate of normal myocardial cells and maintain rhythm stability. In contrast, the *CAV3*‐CMs showed a significant decrease in the beating frequency without fenoterol. Only seven beating events were detected within 150 s (BPM: 2.8), and the beating intervals showed a high degree of variation (CV: 22.73%), presenting a clear irregularity in the beating intervals, that is, a disordered rhythm. Meanwhile, the beating frequency of *CAV3*‐CMs with fenoterol increased to 11 times within 150 s (BPM: 4.4), but CV slightly increased (31.42%). Moreover, the beating frequency of *CAV3*‐CMs with fenoterol was still significantly lower than that of the control group treated with fenoterol, and the CV value was much higher than that of the control group after fenoterol administration, suggesting that the irregular rhythm phenotype of *CAV3*‐CMs did not fully recover under the stimulation of fenoterol (Figure 6).

## 4. Discussion

This study reported a patient with a pathogenic *CAV3* c.136G>A (p.Ala46Thr) mutation in the N‐terminal scaffolding domain, and the long‐term clinical phenotype of this patient was limited to skeletal muscle involvement. The patient initially presented with distal myopathy at 18 years of age, and over the course of 11 years of follow‐up, there was no change in the skeletal muscle symptoms and no self‐reported changes in cardiac structure or function. However, in‐depth analyses using the patient‐specific iPSC‐CMs model uncovered evident structural, functional, and molecular impairments in CMs at the subclinical level. Notably, the CAV3 protein displayed abnormal aggregation in and around the nucleus with nearly complete loss of membrane location. Concomitantly, the basal beating frequency of CMs was significantly decreased, and the rhythm was disordered; the response of these cells to fenoterol, a *β*2‐adrenergic receptor agonist, was markedly weakened. The above findings are the first to show at the level of cellular function that under the clinical manifestation of no cardiac symptoms in the *CAV3* p.Ala46Thr mutation, there are, in fact, detectable CM function abnormalities. This provides important functional evidence supporting the established conclusion that *CAV3* mutations can impair both skeletal muscle and cardiac function, particularly for mutations within the N‐terminal scaffolding domain, and points out the possibility of a huge separation between clinical assessment and cell phenotype.

The abnormal localization of CAV3 protein in the patient′s iPSC‐CMs provides an important molecular mechanism clue for exploring the subclinical functional damage. *CAV3* N‐terminal scaffolding domain acts as the main domain responsible for protein homooligomerization and interactions with signaling molecules [14]. Mutation of the Ala46 residue in the highly conserved sequence in this region most likely interferes with the normal oligomerization of CAV3 directly [15]. This process disorder makes the mutant protein to fail to successfully target the cell membrane, and it abnormally remains in the perinuclear and nuclear areas, affecting the small pits structure on the membrane [16]. As an important signaling hub, caveolae directly regulate several basic physiological processes of CMs. On the one hand, a portion of adrenergic receptor signaling components is localized within caveolae. Therefore, the loss of CAV3 at the plasma membrane may disrupt the anchoring and signaling of these caveolae‐localized adrenergic receptor pathways, which could explain the poor response to fenoterol observed in the mutant cells. On the other hand, caveolar structure is important for the regulation of calcium ion channels. If there is an anomaly, it may disturb the accurate timing of calcium transients, thereby causing the slow and unsteady rhythm [17]. Moreover, CAV3 that abnormally accumulates in the nucleus may also disrupt nuclear transcriptional homeostasis and cause additional damage to cellular function by another route.

These molecular pathological changes confirm the presence of cellular dysfunction in the absence of clinical manifestations. First, compensatory mechanisms are present in vivo, and the other members of the caveolin protein family, such as CAV1/CAV2, can also partially substitute for the function of CAV3 [18]. Secondly, there is a window period during the course of the disease, and the iPSC‐CMs model can reflect the earliest, cell‐intrinsic functional disorders of the disease, whereas clinically significant cardiac structural remodeling or electrophysiological events typically take years or even decades of pathological accumulation to become evident [19]. This is consistent with the pathological progression of many hereditary cardiomyopathies. It also means that phenotypes caused by *CAV3* mutations should be considered as a dynamic continuous disease spectrum rather than independent types [20]. The p.Ala46Thr mutation in this study manifests as subclinical myocardial damage, which may represent the early end of the subclinical‐to‐overt cardiac phenotype continuum. Furthermore, it might advance toward an overt cardiac phenotype with age or the addition of other factors.

Although this study focuses on mechanistic exploration, these above findings also provide insights into potential therapeutic strategies for future interventions. Considering the monogenic pathogenicity of *CAV3* mutation, theoretically, gene replacement therapy to correct the *CAV3* mutation by delivering wild‐type *CAV3* gene via an AAV vector is a feasible approach [21]. Subsequent studies can utilize this model for gene complementation assays in order to directly ascertain whether it has the functional rescue effect, accumulating preclinical evidence for future precise interventions.

The observation of definite CM functional damage in the patient‐specific iPSC‐CMs model of this study has direct clinical implications. Even though the current cardiac imaging and electrophysiological examination results of carriers are normal, they should be redefined as preclinical high‐risk individuals of *CAV3*‐related cardiomyopathy based on their cellular functional defects. This risk stratification helps avoid overlooking the absence of clinical cardiac lesions, which might otherwise mask underlying subclinical dysfunction, and forms a basis for the early monitoring and preventive treatment. In accordance with the relevant domestic and foreign consensuses on the management of cardiomyopathies, for such persons who are carriers of pathogenic mutations but have no clinical phenotype, it is suggested to follow up the heart on a regular basis in the long term [22]. Follow‐up should include ECG, Holter monitoring, and cardiac imaging studies (such as echocardiography) to allow for timely intervention at the early stage of potential functional or structural changes [23].

At the same time, the deficiencies of this study should be objectively acknowledged. First, there are certain deficiencies in the clinical data. Because of the practical conditions, the long‐term follow‐up of the proband and his family was mainly conducted through telephone, and objective cardiac examination data (such as serial ECGs and echocardiography) during the follow‐up period could not be systematically collected, thus limiting the accurate assessment of the dynamic changes of clinical phenotypes. It is worth noting that family member II‐11 died because of a suspected cardiac cause. Although this information has not been confirmed by medical records, it holds significant warning value in clinical genetic counseling, given that sudden death is one of the core phenotypes of hereditary cardiomyopathy. The value of this study lies in the discovery of the potential pathogenic mechanism of this mutation through cell models, thereby providing a mechanism‐driven monitoring direction for subsequent clinical follow‐up. Second, as for the research subjects, this study is a single‐center, small‐sample report mainly involving only one proband and his family, and the results still require validation in a larger group of *CAV3* N‐terminal mutation carriers to determine the actual penetrance and phenotypic manifestations of cardiac injury caused by these mutations. Third, the absence of gene‐edited isogenic controls remains a limitation that can be addressed by using gene editing technology to construct cell lines with the same genes as controls to precisely distinguish genotypes from phenotypes. Fourth, the iPSC‐CM used in this study is still in the early stage of development and lacks the maturity found in mature in vivo cardiac muscle. Nevertheless, despite their immaturity, iPSC‐CMs offer a valuable human cell‐based platform for uncovering early molecular and functional consequences of genetic changes, which cannot be easily studied in primary adult cells. Moreover, future studies could incorporate more comprehensive electrophysiological and contractile assessments to further elucidate the functional consequences of *CAV3* variants and thoroughly investigate the disruption of upstream and downstream protein interactions caused by abnormal CAV3 localization, thereby providing a theoretical basis for targeted intervention.

## 5. Conclusion

Using a patient‐specific iPSC‐CMs model harboring the *CAV3* p.Ala46Thr mutation, we demonstrate for the first time that this mutation can cause the perinuclear aggregation and abnormal membrane localization of CAV3 protein at the cell level and further lead a series of altered spontaneous activity and calcium‐handling behavior, such as slow and disordered automatic rhythm and decreased *β*‐adrenergic responsiveness in CMs. Collectively, these results indicate that the p.Ala46Thr mutation causes subclinical CM dysfunction prior to the onset of overt cardiac clinical manifestations. Clinical assessment and cellular function verification show us through this research that, no matter how limited it is to just presenting with skeletal muscle involvement, people who have pathogenic mutations like p.Ala46Thr in the *CAV3* N‐terminus scaffold region have some CM function risk. Therefore, such patients can be reassigned to a population with a potential risk of heart disease, and it is recommended to conduct lifelong regular heart monitoring. These findings also provide a robust experimental basis for early risk stratification and precise management of *CAV3*‐related disorders while enhancing our understanding of the genetic heterogeneity and phenotypic continuity of such mutations.

## Author Contributions

Junyi Wang, Xintong Zhu, and Yaqiong Li contributed equally to this work.

## Funding

This study was funded by the National Natural Science Foundation of China (10.13039/501100001809; 82302087, 82304050) and the Natural Science Foundation of Chongqing Municipality (10.13039/501100005230; CSTB2023NSCQ‐MSX1057, stc2018jcyjAX0641).

## Conflicts of Interest

The authors declare no conflicts of interest.

## Data Availability

The data that support the findings of this study are available from the corresponding author upon reasonable request.

## References

[1] Tang Z. L. , Scherer P. E. , Okamoto T. , Song K. , Caryn Chu D. , Kohtz S. , Nishimoto I. , Lodish H. F. , and Lisanti M. P. , Molecular Cloning of Caveolin-3, a Novel Member of the Caveolin Gene Family Expressed Predominantly in Muscle, Journal of Biological Chemistry. (1996) 271, no. 4, 2255–2261, 10.1074/jbc.271.4.2255, 8567687. 8567687

[2] Lamaze C. , Tardif N. , Dewulf M. , Vassilopoulos S. , and Blouin C. M. , The Caveolae Dress Code: Structure and Signaling, Current Opinion in Cell Biology. (2017) 47, 117–125, 10.1016/j.ceb.2017.02.014, 28641181. 28641181

[3] Gazzerro E. , Bonetto A. , and Minetti C. , Caveolinopathies: Translational Implications Of Caveolin-3 In Skeletal And Cardiac Muscle Disorders, Handbook of Clinical Neurology: Muscular Dystrophies, 2011, Elsevier, 10.1016/b978-0-08-045031-5.00010-4. 21496630

[4] Vatta M. , Ackerman M. J. , Ye B. , Makielski J. C. , Ughanze E. E. , Taylor E. W. , Tester D. J. , Balijepalli R. C. , Foell J. D. , Li Z. , Kamp T. J. , and Towbin J. A. , Mutant Caveolin-3 Induces Persistent Late Sodium Current and Is Associated With Long-Qt Syndrome, Circulation. (2006) 114, no. 20, 2104–2112, 10.1161/circulationaha.106.635268, 17060380. 17060380

[5] Scalco R. S. , Gardiner A. R. , Pitceathly R. D. , Hilton-Jones D. , Schapira A. H. , Turner C. , Parton M. , Desikan M. , Barresi R. , Marsh J. , Manzur A. Y. , Childs A. M. , Feng L. , Murphy E. , Lamont P. J. , Ravenscroft G. , Wallefeld W. , Davis M. R. , Laing N. G. , Holton J. L. , Fialho D. , Bushby K. , Hanna M. G. , Phadke R. , Jungbluth H. , Houlden H. , and Quinlivan R. , CAV3 Mutations Causing Exercise Intolerance, Myalgia And Rhabdomyolysis: Expanding The Phenotypic Spectrum Of Caveolinopathies, Neuromuscular Disorders. (2016) 26, no. 8, 504–510, 10.1016/j.nmd.2016.05.006, 27312022. 27312022

[6] Huo J. , Mo L. , Lv X. , Du Y. , and Yang H. , Ion Channel Regulation in Caveolae and Its Pathological Implications, Cells. (2025) 14, no. 9, 10.3390/cells14090631, 40358155. PMC12071496 40358155

[7] Markandeya Y. S. , Gregorich Z. R. , Feng L. , Ramchandran V. , O′Hara T. , Vaidyanathan R. , Mansfield C. , Keefe A. M. , Beglinger C. J. , Best J. M. , and Kalscheur M. M. , Caveolin-3 And Caveolae Regulate Ventricular Repolarization, Journal Of Molecular And Cellular Cardiology. (2023) 177, 38–49, 10.1016/j.yjmcc.2023.02.005, 36842733. PMC10065933 36842733

[8] Catteruccia M. , Sanna T. , Santorelli F. M. , Tessa A. , Di Giacopo R. , Sauchelli D. , Verbo A. , Monaco M. L. , and Servidei S. , Rippling Muscle Disease and Cardiomyopathy Associated With a Mutation in the Cav3 Gene, Neuromuscular Disorders. (2009) 19, no. 11, 779–783, 10.1016/j.nmd.2009.08.015, 19773168. 19773168

[9] Fulizio L. , Nascimbeni A. C. , Fanin M. , Piluso G. , Politano L. , Nigro V. , and Angelini C. , Molecular and Muscle Pathology in a Series of Caveolinopathy Patients, Human Mutation. (2005) 25, no. 1, 82–89, 10.1002/humu.20119, 15580566. 15580566

[10] Herrmann R. , Straub V. , Blank M. , Kutzick C. , Franke N. , Jacob E. N. , Lenard H. G. , Kröger S. , and Voit T. , Dissociation of the Dystroglycan Complex in Caveolin-3-Deficient Limb Girdle Muscular Dystrophy, Human Molecular Genetics. (2000) 9, no. 15, 2335–2340, 10.1093/oxfordjournals.hmg.a018926, 11001938. 11001938

[11] Sundblom J. , Stålberg E. , Österdahl M. , Rücker F. , Montelius M. , Kalimo H. , Nennesmo I. , Islander G. , Smits A. , Dahl N. , and Melberg A. , Bedside Diagnosis of Rippling Muscle Disease in Cav3 P.A46t Mutation Carriers, Muscle & Nerve. (2010) 41, no. 6, 751–757, 10.1002/mus.21589, 20229577. 20229577

[12] Vo Q. D. , Nakamura K. , Saito Y. , Akagi S. , Miyoshi T. , and Yuasa S. , Induced Pluripotent Stem Cells in Cardiomyopathy: Advancing Disease Modeling, Therapeutic Development, and Regenerative Therapy, International Journal of Molecular Sciences. (2025) 26, no. 11, 10.3390/ijms26114984, 40507801. PMC12155407 40507801

[13] Richards S. , Aziz N. , Bale S. , Bick D. , Das S. , Gastier-Foster J. , Grody W. W. , Hegde M. , Lyon E. , Spector E. , Voelkerding K. , Rehm H. L. , and ACMG Laboratory Quality Assurance Committee , Standards and Guidelines for the Interpretation of Sequence Variants: A Joint Consensus Recommendation of the American College of Medical Genetics and Genomics and the Association for Molecular Pathology, Genetics in Medicine. (2015) 17, no. 5, 405–424, 10.1038/gim.2015.30, 25741868. PMC4544753 25741868

[14] Park H.-J. , Jang J. , Ryu K.-S. , Lee J. , Lee S.-H. , Won H.-S. , Kim E.-H. , Seo M.-D. , and Kim J.-H. , Structural Interplays in the Flexible N-Terminus and Scaffolding Domain of Human Membrane Protein Caveolin 3, Membranes. (2021) 11, 10.3390/membranes. PMC7912387 33499357

[15] Gazzerro E. , Sotgia F. , Bruno C. , Lisanti M. P. , and Minetti C. , Caveolinopathies: From the Biology of Caveolin-3 to Human Diseases, European Journal of Human Genetics. (2010) 18, no. 2, 137–145, 10.1038/ejhg.2009.103, 19584897. PMC2987183 19584897

[16] Coraspe G. , Andrés J. , Weis J. , Anderson M. E. , Münchberg U. , Lorenz K. , Buchkremer S. , Carr S. , Zahedi R. P. , Brauers E. , Michels H. , Sunada Y. , Lochmüller H. , Campbell K. P. , Freier E. , Hathazi D. , and Roos A. , Biochemical and Pathological Changes Result From Mutated Caveolin-3 in Muscle, Skeletal Muscle. (2018) 8, no. 1, 10.1186/s13395-018-0173-y, 30153853. PMC6114045 30153853

[17] Zaza A. and Grandi E. , Mechanisms of Cav3-Associated Arrhythmia: Protein or Microdomain Dysfunction?, International Journal of Cardiology. (2020) 320, 97–99, 10.1016/j.ijcard.2020.06.051, 32634502. PMC7577955 32634502

[18] Benzoni P. , Gazzerro E. , Fiorillo C. , Baratto S. , Bartolucci C. , Severi S. , Milanesi R. , Lippi M. , Langione M. , Murano C. , and Meoni C. , Caveolin-3 and Caveolin-1 Interaction Decreases Channel Dysfunction Due to Caveolin-3 Mutations, International Journal of Molecular Sciences. (2024) 25, no. 2, 10.3390/ijms25020980, 38256054. PMC10816214 38256054

[19] Joshi J. , Albers C. , Smole N. , Guo S. , and Smith S. A. , Human Induced Pluripotent Stem Cell-Derived Cardiomyocytes (Ipsc-Cms) for Modeling Cardiac Arrhythmias: Strengths, Challenges and Potential Solutions, Frontiers in Physiology. (2024) 15, 1475152, 10.3389/fphys.2024.1475152. PMC11424716 39328831

[20] Hayashi T. , Arimura T. , Ueda K. , Shibata H. , Hohda S. , Takahashi M. , Hori H. , Koga Y. , Oka N. , Imaizumi T. , Yasunami M. , and Kimura A. , Identification and Functional Analysis of a Caveolin-3 Mutation Associated With Familial Hypertrophic Cardiomyopathy, Biochemical and Biophysical Research Communications. (2004) 313, no. 1, 178–184, 10.1016/j.bbrc.2003.11.101. 14672715

[21] Grisorio L. , Bongianino R. , Gianeselli M. , and Priori S. G. , Gene Therapy for Cardiac Diseases: Methods, Challenges, and Future Directions, Cardiovascular Research. (2024) 120, no. 14, 1664–1682, 10.1093/cvr/cvae207. 39302117

[22] Couto J. F. and Martins E. , Recommendations for the Management of Cardiomyopathy Mutation Carriers: Evidence, Doubts, and Intentions, Journal of Clinical Medicine. (2023) 12, no. 14, 10.3390/jcm12144706, 37510821. PMC10380898 37510821

[23] Song L. and Hui R. , Chinese Guideline for the Management of Cardiomyopathies, Chinese Circulation Journal. (2025) 40, no. 5, 420–462, 10.3969/j.issn.1000-3614.2025.05.002.

